# Integrated identification, qualification and quantification strategy for pharmacokinetic profile study of Guizhi Fuling capsule in healthy volunteers

**DOI:** 10.1038/srep31364

**Published:** 2016-08-16

**Authors:** Yun-Xi Zhong, Xiao-Liang Jin, Shi-Yin Gu, Ying Peng, Ke-Rong Zhang, Bing-Chen Ou-Yang, Yu Wang, Wei Xiao, Zhen-Zhong Wang, Ji-Ye Aa, Guang-Ji Wang, Jian-Guo Sun

**Affiliations:** 1 Key Lab of Drug Metabolism and Pharmacokinetics, State Key Laboratory of Natural Medicines, China Pharmaceutical University, Nanjing, Jiangsu, China; 2 China Application Center, SCIEX, Beijing, China; 3 State Key Lab of New-tech for Chinese Medicine Pharmaceutical Process, Jiangsu Kanion Pharmaceutical Co. Ltd, Lianyungang, Jiangsu, China

## Abstract

Guizhi Fuling capsule (GZFL), a traditional Chinese medicine formulation, is widely used in China to relieve pain from dysmenorrhea and is now in a Phase II clinical trial in the USA. Due to the low exposure of the five main medicative ingredients (amygdalin, cinnamic acid, gallic acid, paeoniflorin and paeonol) of GZFL in human, a strategy was built to qualitatively and quantitatively identify the possible metabolites of GZFL and to describe the pharmacokinetic profiles of GZFL in human. In this strategy, LC-Q-TOF/MS was used to identify and structurally elucidate the possible metabolites of GZFL *in vivo*; and a time-based metabolite-confirming step (TBMCs) was used to confirm uncertain metabolites. The simultaneously quantitation results by LC-MS/MS showed low exposure of the five medicative ingredients. According to the strategy we built, a total of 36 metabolites were found and structurally elucidated. The simultaneously semi-quantitative analysis by LC-MS/MS showed that obvious time-concentration curves could be established for 12 of the metabolites, and most of them showed a relatively higher exposure. This study provides a better understanding of the metabolic processes of GZFL in human.

Primary dysmenorrhea (PrD) often occurs during the menstrual period and is associated with abdominal pain, waist pain, dizziness and gastrointestinal discomfort. Epidemiological investigation statistics show that about 13.5% Chinese women suffer from severe PrD which affects women’s normal work schedules and quality of life^1^. Traditional Chinese medicines (TCMs) has an advantage in the treatment of PrD, with its multiple target mechanisms, significant therapeutic effects and mild side effects^2^,^3^,^4^. Guizhi Fuling capsule (GZFL) is one of the most widely used traditional Chinese medical formulation for the treatment of PrD in China, and has shown significant clinical therapeutic effects^5^,^6^,^7^,^8^,^9^,^10^. The prescription of GZFL is a mixture of *cassia twig*, *poria cocos*, *peach kernel*, *cortex moutan radices* and *radix paeoniae alba* of the same quality, and exerts multiple effects, such as promoting blood circulation to remove blood stasis and eliminating disorders, and has been used to treat chronic pelvic inflammatory disease (CPID), hysteromyoma, PrD, endometriosis (EMs) and several other congestion syndromes. GZFL has already finished a Phase II clinical trial in the USA (NCT01588236).

Analysis of TCMs has been regarded as “complex system research”^11^ due to the diverse physical and chemical properties of components from different classes, which may obstruct component analysis^12^,^13^. The low concentrations and exposures of the main medicative ingredients^14^,^15^,^16^,^17^,^18^,^19^,^20^,^21^,^22^,^23^,^24^,^25^,^26^ (gallic acid (GA), cinnamic acid (CA), paeoniflorin (PAE), amygdalin (AMY) and paeonol (PA)) in GZFL also makes it difficult for the study of GZFL *in vivo*. Therefore, the metabolites of the medicative ingredients of GZFL may be a good choice for the pharmacokinetic study instead of the medicative ingredients. The structures of the medicative ingredients are shown in Fig. 1.

In recent years, much analytical equipment, such as capillary electrophoresis (CE), gas chromatography (GC)^27^, and high-performance liquid chromatography (HPLC) combined with mass spectrometry (MS)^28^,^29^,^30^,^31^, has been used to analyze complex components in TCMs^13^. Among this equipment, the most attractive and most promising tool is LC–MS due to its advanced selectivity and sensitivity. Based on this new technology, a strategy has been developed to study GZFL *in vivo*. The strategy contains the following steps: (1) data acquisition, (2) metabolite identification, (3) metabolite structural elucidation, (4) *in vivo* semi-quantitation (5) and *in vivo* metabolites pharmacokinetic profiles study.

Therefore, in order to describe the process of GZFL *in vivo* and to clarify the material basis of the therapeutic effects of GZFL, the pharmacokinetics of medicative ingredients^14^,^15^,^16^,^17^,^18^,^19^,^20^,^21^,^22^,^23^,^24^,^25^,^26^ in GZFL are studied, and the metabolites of GZFL in human plasma after oral administration are profiled. These results contribute to a better understanding of the *in vivo* exposure of GZFL in human body to support further drug development and clinical application.

## Results

### Pharmacokinetics of the medicative ingredients of GZFL in human plasma

After 2 or 3 capsules (contents: AMY 5.66 mg/capsule, CA 0.23 mg/capsule, GA 2.20 mg/capsule, PAE 6.99 mg/capsule and PA 4.79 mg/capsule) of GZFL were orally administered to the 10 subjects, the concentrations of AMY, CA, GA, PAE and PA in the plasma at different time points were detected by LC-MS/MS. After oral administration, the concentration of CA was high, the concentrations of GA and PAE were low and AMY and PA were barely detectable in the plasma. The pharmacokinetic study results indicated that the drug concentration in the plasma increased with increasing dose. The T_max_ of the drugs was approximately 1–3 h, and the t_1/2_ of the drugs was approximately 0.90–4.34 h. The pharmacokinetic parameters of CA, GA and PAE are shown in Table 1. (The pharmacokinetic parameters of AMY and PA are not shown because they are beyond computation.). The ions and parameters for detection are shown in Table 2.

### A strategy proposed to describe the possible global pharmacokinetic profiles of GZFL *in vivo*

A strategy was designed to identify, qualify and quantify possible metabolites to describe the global pharmacokinetic profiles of GZFL *in vivo*. This strategy for the *in vivo* study of GZFL included the following steps: (1) Global data acquisition of the samples from different time points by LC-Q-TOF/MS; (2) Mass peak mining using software, such as MetabolitePilot 1.5 and PeakView, to analyze the sample data to identify potential metabolites; (3) Metabolite structural elucidation by targeted mass fragmentation; (4) Semi-quantitation and PK profile study of the metabolites through the MRM mode of LC-MS/MS; and (5) Study of the metabolic progress of GZFL *in vivo* through the metabolites.

#### Metabolite peak mining *in vivo*

The data from LC-Q-TOF/MS contain the entire package of mass information to be discovered using a suitable method, as with mineral deposits. To obtain useful information from the deposit, PeakView software was mainly used for metabolite mining. In this software, a sample after drug administration and a blank sample are compared directly to find all of the compounds in both of the samples, which usually numbered in thousands. Then, the data are manually filtered to narrow the targeted peaks to less than one hundred by setting the ratio of sample to control to 10 and to obtain the possible metabolites that are needed. This step contained the following factors: (1) retention time, (2) change in mass defect, (3) intensity of the compounds in the sample and blank sample; and (4) error in parts per million (ppm).

#### Metabolite Structural Elucidation

MetabolitePilot 1.5 software uses multiple data-processing algorithms: generic peak finding, multiple mass defect filtering, isotope pattern matching, and finding metabolites based on common product ions or neutral losses. A sample after drug administration and a blank sample are automatically compared to find possible metabolites and metabolic pathways using this software. Information of the metabolites such as name, formula, m/z, ppm, R.T. and peak area will be shown in this software. MetabolitePilot 1.5 software will also give the MS/MS fragments in order to give accurate masses of different parts of the drug for metabolite structural elucidation.

### The time-based metabolite-confirming step (TBMCs)

**Figure 2:**
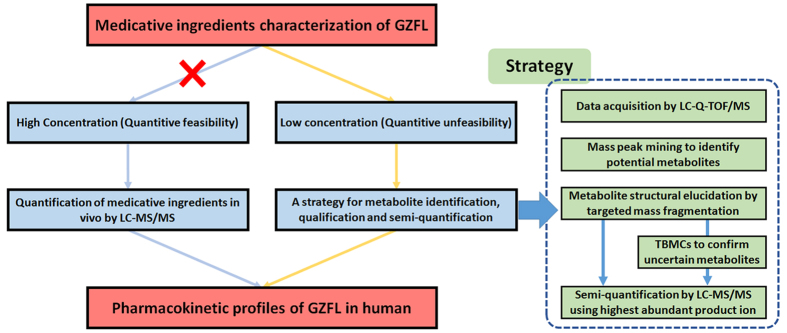
The total strategy proposed to study the pharmacokinetic profiles of GZFL in human. *“TBMCs” stands for “time-based metabolite confirming steps”.

This step is based on the well-known pharmacokinetic theory that every molecule that enters the body will follow the ADME process. If an untargeted peak shows a well-defined PK profile after drug administration, it is assumed to be related to the drug. Therefore, in this step, the parent ions and product ions of the uncertain metabolites will be chosen for the detection with LC-MS/MS for the pharmacokinetic profile study. The overall strategy is shown in Fig. 2.

### Identification and qualification of the metabolites of GZFL in human plasma

The identification and qualification of the metabolites of GZFL in human plasma is a critical step to describe the global PK profiles of GZFL in human. LC-Q-TOF/MS was used for the identification of biological metabolites in human plasma. Due to the complex ingredients in GZFL, metabolite identification and qualification are very difficult. Using the strategy we built above, a total of 36 different possible metabolites were identified as shown in Table 3. AMY, CA, GA, PA and PAE metabolized into 3, 2, 7, 15, 3 metabolites respectively. Metabolite M31 to M36 were found based on TBMCs in human plasma.

### Structural elucidation of the metabolites of GZFL in human plasma

**Figure 3:**
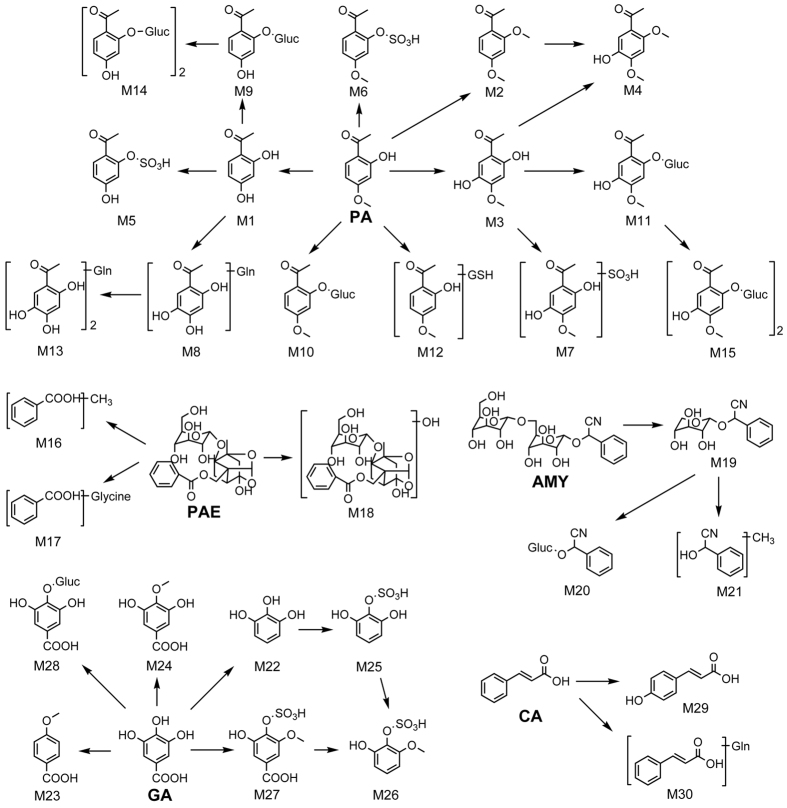
The metabolic pathway of AMY, CA, GA, PA and PAE in human plasma.

In this part, according to the calculations of PeakView and MetabolitePilot 1.5 software and the speculation and comparison of MS/MS fragments, some of the structures and pathways were deduced. The specific structures and metabolic pathways are shown in Fig. 3.

### Semi-Quantitative results of the metabolites from LC-MS/MS

**Figure 4:**
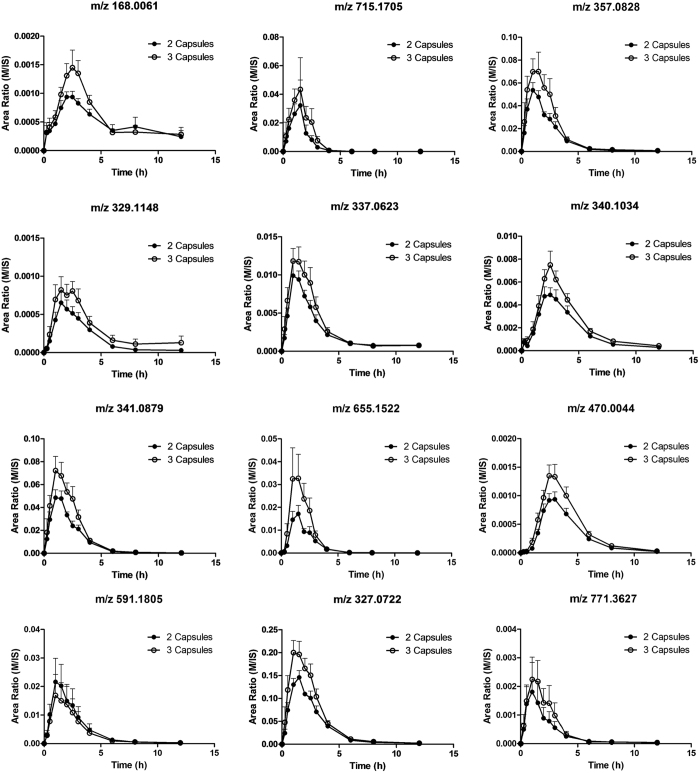
Time-response (Area ratio) curves of 12 representative metabolites of GZFL in human plasma.

The molecular ions of each metabolite given by TOF/MS were chosen as Q1, and the largest product ion from all of the product ions of each metabolite that was found by LC-Q-TOF/MS were chosen as Q3, and the values of DP and CE were optimized by comparing different offsets. The ions and parameters chosen for this detection are shown in Table 2. The time-concentration curves of 12 metabolites are listed in Fig. 4. Based on the curves, the metabolites of GZFL in human plasma were semi-quantified and then used to speculate the process of GZFL *in vivo*. There was an apparent dose relationship between the metabolite profiles after two or three capsules were taken.

## Discussion

There are two challenges when studying on pharmacokinetics of TCMs. One is the difficulty to build a simple and rapid detection method for the detection of complex components from different classes in TCMs simultaneously; and the other one is the difficulty to characterize pharmacokinetic process of TCMs by medicative ingredients *in vivo* due to the low exposure, which might result from the low concentration in medicine materials or the low oral bioavailability of prototypes. In this experiment, an LC-MS/MS method was set up to simultaneously quantitate the five commonly recognized medicative ingredients (AMY, CA, GA, PA, and PAE) which represent the components of GZFL (*cassia twig*, *poria cocos*, *peach kernel*, *cortex moutan radices*, and *radix paeoniae alba*) and the newly found 12 metabolites. The specificity, recovery, matrix effect, accuracy, precision, linearity, and stability of this method were validated (data to be published otherwise). The RSD of the recovery of PAE was 2.7–10.6%, and the RSDs of matrix effects of CA and PAE were 6.3–13.4% and 1.7–10.4%. The stability results showed that GA was not stable when kept at room temperature for 24 h in human plasma (9.3–36.7%). The stability of GA kept at room temperature for 6 h in human plasma was further validated, and the results showed that GA was stable at room temperature for 6 h in human plasma (93.1–114.0%). The results indicated that after harvesting the plasma sample in clinic, all of the samples should either be kept in a −70 °Cfreezer or extracted within 6 h to maintain the stability of GA.

Only CA, GA and PAE could be detected in human plasma, whereas AMY and PA could not be detected because of their low exposure. The C_max_ of CA was greater than 100 ng/ml, but the reports of the pharmacological activities of CA mainly dealt with antitumor activity, hypoglycemic activity, hypolipidemic activity and antibacterial and anti-inflammatory activity, not with relieving dysmenorrhea^24^,^32^. GA and PAE had the effect of relaxing smooth muscle and relieving pain^21^,^33^, but their C_max_ values were both less than 10 ng/ml. Therefore, the efficacy of GZFL was likely not directly due to the supposed medicative ingredients (AMY, CA, GA, PA, and PAE) but to their metabolites.

We further built a strategy for identification, qualification and quantification of metabolites of GZFL in human plasma. In this strategy, an LC-Q-TOF/MS high-resolution mass spectrometer was used to identify the metabolites of GZFL in human plasma. Using software and our strategy, a total of 36 probable metabolites were found. The identification and qualification of metabolites was based on the chromatographic peaks in plasma samples and the concentration-time curves. The structural elucidation was carried out with the help of MetabolitePilot 1.5 software and the analysis of product ions from parent drugs and metabolites. PA was found to be metabolized into 15 metabolites, with the fragment ions m/z 151.08, speculated as demethylation fragmentation, detected in M1, M4, M5, M9, M13 and M14. The demethyl metabolite showed the fragment ion m/z 327.07 after further combination with glucuronic acid, and m/z 327.07 can dimerize to form m/z 655.15. Conjugation with sulfuric acid and glutathione was also detected. PAE can be metabolized into 3 metabolites, and the glucoside structure is easily hydrolyzed into glycosyl and aglycone, and a p-hydroxylation metabolite was also detected. AMY was metabolized into 3 metabolites. The glucoside bond in AMY was hydrolyzed to form metabolites. GA was metabolized into 7 metabolites. The conjugations of GA with glucuronic acid, sulfuric acid and glutathione on phenolic hydroxyl were also detected, and CA was metabolized into 2 metabolites, a p-hydroxylation metabolite and a glucuronic acid conjugate, respectively. According to former ion fragment speculation, most of the biotransformed metabolites in plasma were glucuronides and sulfuric acid conjugates derived from PA, PAE, AMY, GA and CA. Six probable metabolites among the 36 metabolites could not be attributed to their parent drugs.

The advantage of qualitative detection by LC-Q-TOF/MS, high-resolution mass spectrometer and the quantitative detection by LC-MS/MS was used for semi-quantitative analysis of several representative metabolites from the 36 metabolites to study the metabolites in human. A total of 12 metabolites shows apparent time-plasma concentration curves in Fig. 4. The results above provide evidence supporting the existence of metabolites that we obtained from the LC-Q-TOF/MS high-resolution mass spectrometer. Moreover, some substances that were found by PeakView showed apparent time-concentration curves in human plasma, and although the structures and sources of those substances were not determined, they were hypothesized to be metabolites as well.

The concentration of metabolites in human plasma cannot be quantitatively determined due to the lack of authentic standards. Three PA-glucuronide metabolites showed the highest intensity based on peak area. Due to the lack of activity of PA spiked in rat plasma, these three PA-glucuronide metabolites were speculated as the effective components such that further pharmacological research should be done after synthesis or isolation of three PA-glucuronide metabolites.

The metabolites summarized in Table 3 were identified by our strategy, qualitatively detected by LC-Q-TOF/MS and quantitatively determined by LC-MS/MS. Compared with published strategies^12^,^17^,^27^,^31^,^34^, the identification strategy is more suitable to identifying TCMs metabolites due to its accuracy and simplicity. Some trace metabolites, which often exist in TCMs metabolism, can be identified by this strategy. Further verified experiments should be performed to identify the correction of metabolite structures.

The elucidation of the pharmacological mechanisms of TCMs has thus far been challenging. Thus, to clarify the pharmacological mechanism of GZFL, metabolite identification, qualification and quantitation is necessary. The identification of active metabolites or specialized metabolic pathways may provide a better understanding of the *in vivo* metabolic processes of GZFL to provide new ideas to explore this mechanism and to provide a theoretical foundation for hypotheses. The identification of GZFL metabolites in this experiment can achieve this goal.

## Methods

### Reagents and chemicals

AMY, CA, GA, PAE, PA and GZFL (KYG0395, 0.465 g/capsule, batch NO.: 20111201; the contents: AMY 5.66 mg/capsule, CA 0.23 mg/capsule, GA 2.20 mg/capsule, PAE 6.99 mg/capsule and PA 4.79 mg/capsule) were provided by KANION Pharmaceutical (Jiangsu, China). 4′-Hydroxyacetophenone (internal standard, 99.77%) was provided by Shanghai Institute of Pharmaceutical Industry (Shanghai, China). An Oasis HLB 1cc (30 mg) solid-phase extraction cartridge was purchased from Waters (USA). Ultrapure water, which was used throughout the experiments, was prepared using a Milli-Q Ultrapure water purification system (Millipore, Bedford, USA). All of the other reagents were of HPLC grade or of the highest grade commercially available.

### Clinical study design

#### Study subjects

A total of 10 healthy female subjects took part in this study. None of the subjects had an organic lesion of the reproductive system nor did any of the subjects have their menstrual period during treatment. Ethical approval was granted by the research ethics committees at the Hospital of Integrated Traditional Chinese and Western Medicine in Jiangsu Province (2012LW016, date of registration, October 11, 2012) and conducted under the guidelines of the Helsinki Declaration and the International Conference on Harmonization-Good Clinical Practices (ICH-GCP). After the clinical trial had been clearly explained to the volunteers, written consent was obtained from all subjects. GZFL has already finished a Phase II clinical trial in the USA (NCT01588236, date of registration, April 26, 2012).

The subjects were divided into 2 groups according to weight and were arranged into phase I clinical care units one day before the therapeutic treatment began. After being given unified food once in the afternoon, all of the subjects fasted overnight for 10 h but with free access to water before the test. Then, 1 h after being given a unified breakfast on the test day, one of the groups was given 2 capsules of GZFL, whereas the other group was given 3 capsules of GZFL orally. The test lasted for 36 h, and all of the food was unified, which was arranged by the hospital during the test. No other drugs, smoking, coffee, tea, or drinks that contained alcohol or caffeine were allowed, and all of the subjects remained calm during the test. Cross medication was taken one week later.

5-ml blood samples were withdrawn from subjects and collected in heparinization centrifuge tubes at 0h (before taking medication), 0.25, 0.5, 1, 1.5, 2, 2.5, 3, 4, 6, 8, 12, 24, and 36h after taking the medication. All the blood samples were immediately centrifuged at 3000 g for 5min to obtain plasma. All plasma samples were frozen at −70 °C until analysis.

### Plasma samples preparation

All of the samples were prepared using the solid-phase extraction method. Before extraction, the Oasis HLB 1cc (30 mg) extraction cartridges were activated by 1 mL of methanol and equilibrated with 2 mL of water. Then, 1 mL of plasma was added to 10 μl of IS and mixed with 1 mL of water containing 1% formic acid, and the 2 mL mixtures were loaded onto the extraction cartridges. After being washed with 1 mL of water, 0.5 mL of methanol was added to elute the medicative ingredients. All of the eluent (approximately 0.5 mL) was collected and evaporated to dryness in a rotary evaporator (SPD2010, Thermo Fisher Scientific, NJ, USA) at 45 °C. The residue was reconstituted in 100 μl of reconstitution fluid (20% methanol and 80% water) and centrifuged at 30,000 g for 5 min, and a 5.0 μl aliquot was injected for analysis.

### Identification of the metabolites of the medicative ingredients of GZFL

#### LC-Q-TOF/MS system

The HPLC system consisted of a Shimadzu DGU-20A5 online degasser, two Shimadzu LC-30AD pumps with a high-pressure mixer, a Shimadzu CTO-20A column oven and a Shimadzu SIL-30AC autosampler (Shimadzu, Kyoto, Japan). Chromatographic separation was carried out at 40 °C on an HPLC 100-5C_18_ column (150 × 2.1 mm; Kromasil) with the mobile phase of water containing 0.05% formic acid (A) and acetonitrile (B). Gradient elution started from 5% B for 1 min; increased linearly to 18% B over 2 min, to 40% B over the next 3 min and to 90% B over the next 1 min; was maintained at 90% B for 3 min; decreased linearly to 5% B over the next 0.5 min; and was maintained for 4.5 min to re-equilibrate the column. Mass spectrometric analysis was performed with a LC-Q-TOF/MS high-resolution mass spectrometer (Triple-TOF 5600, Sciex) operating in negative mode using a DuoSpray ion source. High-resolution MS and MS/MS data were acquired by the information-dependent acquisition (IDA) method. The IDA method was composed of a TOF MS survey scan (accumulation time 250 ms) and 4 dependent product ion scans (accumulation time 100 ms). The mass ranges of the TOF MS and product ion scans were both m/z 50–800. The parameters were set as follows: ion source gas 1, 50 psi; ion source gas 2, 60 psi; temperature, 550 °C; curtain gas, 30 psi; ion spray voltage, −4500 V; declustering potential, −120 V; and collision energy, −10 eV in TOF MS and −40 eV in the product ion scans.

### Semi-quantitation of 12 metabolites of GZFL

#### LC-MS/MS system

The HPLC system was the same as described above. Mass spectrometric analysis was performed on a Sciex ADME mass spectrometer (API4000, Sciex) that was equipped with an electrospray ionization (ESI) interface. Vacuum in the mass detector was obtained using a Turbo molecular pump. The MS parameters for ESI were as follows: ion spray voltage, −4500 V; ion source gas 1, 65 psi; ion source gas 2, 60 psi; temperature, 550 °C; curtain gas, 35 psi; collision gas, 10 psi; entrance potential,-10 V; and collision cell exit potential, −15 V. The scan type was multiple reaction monitoring (MRM) in negative ionization mode.

## Additional Information

**How to cite this article**: Zhong, Y.-X. *et al*. Integrated identification, qualification and quantification strategy for pharmacokinetic profile study of Guizhi Fuling capsule in healthy volunteers. *Sci. Rep.* **6**, 31364; doi: 10.1038/srep31364 (2016).

## Figures and Tables

**Figure 1:**
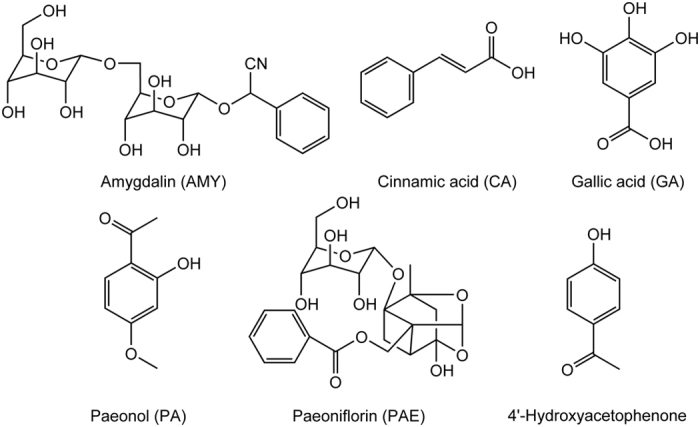
The structures of the medicative ingredients of GZFL and the internal standard (IS, 4′-hydroxyacetophenone).

**Table 1:** The pharmacokinetic parameters of CA, GA and PAE orally administrated 2 and 3 capsules.

Parameters	2 capsules			3 capsules		
	CA	GA	PAE	CA	GA	PAE
C_max_ (ng/ml)	107.24	4.36	3.00	148.15	6.14	3.46
T_max_ (h)	1.0	2.2	3.0	0.9	2.1	2.7
t_1/2_ (h)	1.74	2.73	4.34	1.50	1.66	3.81
MRT (h)	2.55	4.88	7.40	2.25	3.29	6.63
AUC_0-τ_ (ng/ml*min)	144.13	8.62	10.66	210.66	13.44	14.81
AUC_0-∞_ (ng/ml*min)	164.66	12.60	16.94	227.25	15.44	20.70
F*	—	—	—	128.2%	229.8%	155.6%
AUC extrapolation ratio	12.0%	36.2%	33.0%	9.0%	24.0%	32.0%

^*^compared with the parameters orally administrated 2 capsules.

**Table 2:** The mass spectrometric detection parameters of AMY, CA, GA, PAE, PA, IS and 12 metabolites.

Name	Q1 (Da)	Q3 (Da)	Dwell (msec)	DP (V)	CE (eV)
AMY	525.10	121.10	10	−50	−30
CA	146.80	102.80	10	−50	−15
GA	168.80	124.80	10	−67	−20
PAE	456.10	322.90	10	−120	−17
PA	164.80	149.80	10	−72	−21
IS	134.90	91.80	10	−75	−30
M31	168.00	123.05	10	−60	−25
M15	715.17	357.02	10	−60	−25
M11	357.08	166.06	10	−60	−25
M34	329.11	135.09	10	−60	−25
M35	337.06	161.06	10	−60	−25
M33	340.10	161.08	10	−60	−25
M10	341.08	165.09	10	−60	−25
M14	655.15	327.03	10	−60	−25
M12	470.00	357.02	10	−60	−25
M13	591.18	327.09	10	−60	−25
M9	327.07	151.08	10	−60	−25
M36	771.36	357.02	10	−60	−25

**Table 3:** The information of the metabolites of GZFL in human plasma.

Metabolitecode	Parent ionsm/z	t_R_ (min)	Formula	Product Ions m/z	Prototype
M1	151.0402	7.49	C_8_H_8_O_3_	65.0386, 92.0259, 136.1032, 151.0402	PA
M2	179.0714	9.27	C_10_H_12_O_3_	92.0267, 136.0158, 179.0714	PA
M3	181.0514	5.74	C_9_H_10_O_4_	135.0452, 163.0402, 181.0514	PA
M4	195.0665	8.23	C_10_H_12_O_4_	93.0326, 119.0494, 121.0316, 135.0456, 151.0755, 195.0665	PA
M5	230.9956	7.16	C_8_H_8_O_6_S	230.99, 151.04, 135.04, 109.03	PA
M6	245.0489	12.71	C_9_H_10_O_6_S	165.0921, 245.0489	PA
M7	261.0061	12.64	C_9_H_10_O_7_S	261.01, 181.05, 165.03, 135.03, 122.03	PA
M8	295.0973	9.18	C_13_H_16_N_2_O_6_	103.0545, 147.0457, 251.0968, 295.0973	PA
M9	327.0722	7.49	C_14_H_16_O_9_	85.0291, 113.0240, 151.0398, 175.0243, 327.0722	PA
M10	341.0879	7.72	C_15_H_18_O_9_	113.0256, 150.0323, 165.0555, 275.0433, 341.0903	PA
M11	357.0828	7.69	C_15_H_18_O_10_	59.0134, 113.0240, 166.0272, 175.0247, 181.0505, 357.0828	PA
M12	470.0044	6.26	C_19_H_25_N_3_O_9_S	75.0676, 99.0648, 113.0767, 161.0828, 470.0044	PA
M13	591.1805	6.73	C_26_H_32_N_4_O_12_	145.1044, 151.0808, 175.0652, 263.0945, 327.0282, 591.1805	PA
M14	655.1522	7.49	C_28_H_33_O_18_	113.0240, 151.0399, 175.0245, 327.0718, 413.2715, 655.1522	PA
M15	715.1705	7.1	C_30_H_36_O_20_	113.0765, 175.0561, 181.0803, 263.0438, 357.0217, 715.1705	PA
M16	135.0438	7.68	C_8_H_8_O_2_	135.04, 121.03, 77.04	PAE
M17	178.0512	6.69	C_9_H_12_NO_3_	178.05, 121.03, 77.04	PAE
M18	495.1632	7.11	C_23_H_28_O_12_	495.16, 479.16, 327.11, 165.05, 121.03, 77.04	PAE
M19	264.0722	4.11	C_13_H_15_NO_5_	264.08, 132.05, 116.06, 107.06	AMY
M20	308.0781	7.39	C_14_H_15_NO_7_	308.08, 132.05, 116.06, 107.06	AMY
M21	147.0737	12.41	C_9_H_9_NO	355.12, 341.12, 179.06	AMY
M22	125.0250	6.7	C_6_H_6_O_3_	79.0198, 124.0216, 125.0250	GA
M23	151.0427	7.70	C_8_H_8_O_3_	151.04, 107.05, 93.04, 79.02, 55.02	GA
M24	183.0304	5.10	C_8_H_8_O_5_	123.0132, 124.0204, 168.0153,183.0304	GA
M25	204.9810	6.65	C_6_H_6_O_6_S	79.9579, 125.0209, 204.9810	GA
M26	218.9977	7.02	C_7_H_8_O_6_S	79.9580, 124.0209, 139.0461,218.9977	GA
M27	262.9870	12.34	C_8_H_8_O_8_S	124.0213, 168.0155, 183.0405,262.9870	GA
M28	345.0463	2.55	C_13_H_14_O_11_	125.0293, 169.0234, 345.0463	GA
M29	275.1039	7.87	C_14_H_16_N_2_O_4_	91.0531, 109.0399, 112.0368, 127.0506, 147.0472, 275.1039	CA
M30	163.0405	5.74	C_9_H_11_O_2_	117.0560, 135.0454, 163.0405	CA
M31	168.0061	5.20	Unknown	124.0659, 168.0061	Unknown
M32	308.0767	6.72	Unknown	132.0405, 308.0767	Unknown
M33	340.1034	7.27	Unknown	None	Unknown
M34	329.1148	8.74	Unknown	171.1016, 211.1322, 229.1428, 329.1148	Unknown
M35	337.0624	6.27	Unknown	337.06, 161.06	Unknown
M36	771.3673	7.75	Unknown	771.36, 357.02	Unknown
